# The COVID‐19 Pandemic and Intergenerational Support: Results Based on the Health and Retirement Study COVID-19 Module

**DOI:** 10.1093/geroni/igab046.1358

**Published:** 2021-12-17

**Authors:** Zhen Cong, Guanggang Feng

**Affiliations:** 1 University of Texas at Arlington, Arlington, Texas, United States; 2 University of Texas at Arlington, School of Social Work, Texas, United States

## Abstract

This study examined relationships between COVID-19 exposure and intergenerational support patterns. The data was from the 2020 Health and Retirement Study (HRS) COVID-19 Module (N=3266). The latent class analysis (LCA) was used to identify the types of intergenerational support based on respondents’ reports on whether they provided and received financial and instrumental support from either coresident children or non-coresident children. Two classes were identified, namely, the high interaction group and the low interaction group. Logistic regression showed that respondents who had COVID-19 and had increased spending as a result of COVID-19 were more likely to be in the high interaction group. Other types of COVID-19 exposure, i.e., knowing someone being diagnosed or knowing someone who died from COVID-19 were not significant.

